# Overbooking for physical examination considering late cancellation and set-resource relationship

**DOI:** 10.1186/s12913-021-07148-y

**Published:** 2021-11-20

**Authors:** Te-Wei Ho, Ling-Chieh Kung, Hsin-Ya Huang, Jui-Fen Lai, Han-Mo Chiu

**Affiliations:** 1 Department of Surgery, College of Medicine, National Taiwan University, Taipei, Taiwan; 2 Department of Information Management, College of Management, National Taiwan University, Taipei, Taiwan; 3 Health Management Center, National Taiwan University Hospital, Taipei, Taiwan; 4 Department of Internal Medicine, College of Medicine, National Taiwan University, Taipei, Taiwan

**Keywords:** Healthcare management, Physical examination, Late cancellation, Overbooking, Set-resource relationship, Stochastic mathematical programming, Logistic regression, Monte Carlo simulation, Discrete-event simulation

## Abstract

**Background:**

Late cancellations of physical examination has severe impact on the operations of a physical examination center since it is often too late to fill vacancy. A booking control policy that considers overbooking is then one natural solution. Unlike appointment scheduling problems for clinics and hospitals, in which treating a patient mostly requires only one type of resource, a physical examination set typically requires multiple types of resources. Traditional methods that do not consider set-resource relationship thus may be inapplicable.

**Methods:**

We formulate a stochastic mathematical programming model that maximizes the expected net reward, which is the examination revenue minus overage cost. A complete search algorithm and a greedy search algorithm are designed to search for optimal booking limits for all examination sets. To estimate the late cancellation probability for each individual consumer, we apply logistic regression to identify significant factors affecting the probability. After clustering is used to estimate individual probabilities, Monte Carlo simulation is conducted to generate probability distributions for the number of consumers without late cancellations. A discrete-event simulation is performance to evaluate the effectiveness of our proposed solution.

**Results:**

We collaborate with a leading physical examination center to collect real data to evaluate our proposed overbooking policies. We show that the proposed overbooking policy may significantly increase the expected net reward. Our simulation results also help us understand the impact of overbooking on the expected number of customers and expected overage. A sensitivity analysis is conducted to demonstrate that the benefit of overbooking is insensitive to the accuracy of cost estimation. A Pareto efficiency analysis gives practitioners suggestions regarding policy determination considering multiple performance indications.

**Conclusions:**

Our proposed overbooking policies may greatly enhance the overall performance of a physical examination center.

## Background

### Overbooking considering sets and resources

No-shows and late cancellations, which cause healthcare resource waste and longer patient/consumer waiting, are critical issues for healthcare service providers to deal with [1, 2]. It is documented that the no-show rates in physical examination related sectors may be up to 17.6% [3–5]. In a survey with respect to 200 continuity clinics, the mean no-show percentage is 30.9%, where the maximum one is 80% [6]. In fact, among clinic size, no-show probability, and variation of service times, which are the three major environmental factors affecting the performance of a healthcare appointment system, no-show probability is considered the most significant [7]. It is widely suggested that no-shows and late cancellations should be taken into account when designing an appointment management system [8].

For healthcare environments with no-shows and late cancellations, operations researchers have done a lot of works in appointment management to enhance efficiency [9]. To deal with the uncertainty brought by random no-shows and late cancellation, one way is to adopt dynamic appointment scheduling [10–12]. By doing this, a healthcare service provider allocates physicians’ available time slots to patients periodically by considering random appointment requests, no-shows, and late cancellations. While patients’ preference over physicians and/or time slots and delay-dependent no-show probability are the major issues under consideration, the capacity (e.g., number of time slots in each period) is considered fixed. Another way to tackle no-shows and late cancellations is to relax the fixed capacity constraint and adopt overbooking, which has been widely applied in the hotel and airlines industry decades ago [13, 14]. The possibility and potential benefit of overbooking in the healthcare industry are also investigated in recent years [15–23]. Given no-show probabilities of patients and an ideal number of patients for each time slot, these studies aim to determine a booking limit (which is typically larger than the ideal number of patients) to find a balance between clinic revenue, physician overtime, patient waiting, and the general accessibility of medical services.

For the physical examination center we collaborate with, dynamic appointment scheduling is already applied, and overbooking is under consideration. Interestingly, while there are many past works that develop overbooking policies for outpatients requesting clinical services, none of the policies developed in the above works may be directly applied to the physical examination center due to a major difference between these two types of services. For most clinical services, each outpatient typically requires only one type of *resource* (e.g., a physician). When developing an overbooking policy, booking limits are determined directly for resources (e.g., at most five bookings for a specific physician in an hour), and whether *overage* occurs (i.e., more patients than expected show up) is determined also for each resource (e.g., whether a physician needs to work overtime). In short, the determination of booking limits and calculation of overage costs are aligned.

Physical examinations, however, are mostly provided to consumers in the format of *sets*, where an examination set requires multiple resources such as gastroenteroscope, magnetic resonance imaging (MRI), computed tomography scan (CT), etc. For example, our collaborating center offers 22 examination sets for consumers to select from (cf. Table 13 in the appendix for details). Different examination sets require different resources; for example, among the 22 examinations sets, three of them require gastroenteroscope, six require MRI, and two require CT. For most physical examination centers, while overage levels are still calculated based on resources, booking limits are determined for examination sets. In other words, the determination of booking limits and calculation of overage costs are *not* aligned. The fact that the same level of overage at different resources may bring in quite different costs and inefficiency further complicates the problem.

In summary, the relationship between sets and resources, which is called *set-resource relationship* in this study, must be incorporated in developing an overbooking policy for an physical examination center. To the best of our knowledge, this work is the first one that develops a mathematical model of overbooking considering the set-resource relationship. This work may thus contribute to both practice and academia.

### Factors affecting late cancellation probabilities

To generate an overbooking policy in practice, a theoretical model is not enough. The estimation of late cancellation probability based on historical booking and cancellation data is also required.

Many works have been devoted to the investigation of factors explaining patient no-shows, mostly about clinical appointments. Parente et al. [3] classify the explanatory variables of the early works into three groups: (1) factors related to individuals, (2) factors related to environmental conditions, and (3) factors associated with practices designed to increase patient’s attendance rates. Regarding demographic information of patients, it has been reported that age, gender, ethnicity, type of medical insurance, socioeconomic conditions, diseases and conditions, past no-show and late cancellation records are correlated with no-shows and late cancellations [23–26]. For environmental factors, information about office accessibility, transportation convenience, whether childcare is provided, appointment lead time (appointment delay), examination type (specialty group), examination season, and service quality have been shown to be effective [3, 25, 27–29]. Finally, education, penalties, and reminders by phone or email are also documented as useful devices to reduce no-show probabilities [6, 30–33]. For most the works mentioned above, regression-type analysis is the major research tool.

In this study, we follow the above works to search for significant factors that are correlated with late cancellations in the collaborating physical examination center. While the above works provide some guidance, not all variables are available in this study. For example, the center has no information about a consumer’s ethnicity and type of medical insurance. Moreover, as there is only one center in this study, there is no way to examine facility-related factors such as office accessibility and transportation convenience and activities like education and reminders using historical bookings. Table 1 lists the variables we obtain from the collaborating center that have been documented in the literature, including a consumer’s age, a consumer’s gender, appointment lead time (number of days between the examination day and appointment day), season (in which month the examination is conducted), and examination set. We investigate these variables about their significance and use the significant ones to construct our late cancellation probability estimation model.

**Table 1:** Factors comparison between this study and the literature

Variable	Our study	[27]	[28]	[25]	[30]	[26]	[29]	[3]	[23]
Age	S.		v		v	v	v	v	v
Gender	N.S.							v	
Appointment lead time	S.	v	v	v			v		
Season	S.							v	
Examination set (specialty group)	N.S.	v						v	
Group booking	S.								
Holiday	S.								

S. means the variable is statistically significant and N.S. means the variable is not significant in our study.

v means the variable is statistically significant in that study

There are also some variables that are not documented in literature but turn out to be effective in this study. In particular, we find that whether a booking is a group booking, i.e., more than one consumers book for the same day in one phone call or one online submission, and whether a booking is in a national holiday have significant impact on late cancellation probabilities. These variables are also considered to be used in the process of estimating late cancellation probabilities. In other words, this work adds to the literature by reporting new variables that are significantly correlated with late cancellations.

### A comprehensive case study

In this study, we construct a stochastic optimization model to do overbooking by considering the set-resource relationship. We also propose a way of late cancellation probability estimation to generate the estimated probability for each individual consumer. To further create values for physical examination practitioners, we collect real booking and cancellation data from our collaborating physical examination center to conduct a comprehensive case study. We describe the whole process of data cleansing, probability estimation, booking limit optimization, and performance evaluation of various overbooking policies. We believe that the description of the complete process may serve as a reference for practitioners to apply the same idea in their own physical examination centers.

For ease of exposition, in the sequel we will use the term “late cancellations” to include both late cancellations and no-shows.

## Methods

To generate an overbooking policy, there are two major steps: late cancellation probability estimation and booking limit optimization, where the first part is to find parameter values for the second part. Below we will first introduce the stochastic mathematical programming model for booking limit optimization. We will then describe the process of late cancellation probability estimation, which is based on logistic regression and Monte Carlo simulation. While the general process is presented in this section, a detailed case study that may serve as an example may be found in the next section.

### Stochastic mathematical programming

Consider a physical examination center which opens for booking and subject to late cancellations. The center provides various physical examination *sets* to fit different consumers’ needs. Each set includes several *resources*, such as gastroenteroscope, MRI, CT, etc. Let *I*={1,2,...,*n*} be the collection of resources and *J*={1,2,...,*m*} be the collection of examination sets, where *m* is the number of examination sets. The original *booking limit* (numbers of open slots) and price of set *j* are *B*_*j*_ and *P*_*j*_, respectively, for each *j*∈*J*. Let *X*_*j*_ be the random number of consumers that show up in a day to do set *j* with no late cancellation and *x*_*j*_ be a possible realization of *X*_*j*_. The *expected revenue* that the center may earn with the original booking limits may then be calculated as

$$ \sum_{x_{1}=1}^{B_{1}} \sum_{x_{2}=1}^{B_{2}} \dots \sum_{x_{m}=1}^{B_{m}} x_{j} P_{j} \prod \limits_{j \in J} \Pr(X_{j} = x_{j}), $$

where Pr(*X*_*j*_=*x*_*j*_) is the probability for *x*_*j*_ consumers to show up for set *j*.

Knowing that the *B*_*j*_ consumers who booked set *j* may not all show up, the center manager is considering to increase the booking limit to *N*_*j*_ for set *j*. We will call a collection of booking limits *N*_1_,*N*_2_,..., and *N*_*m*_ as a *booking limit combination*, which is to be determined by the center manager. Obviously, in an overbooking policy we have *N*_*j*_≥*B*_*j*_. The expected revenue that the center may earn with the booking limit combination *N*_1_,*N*_2_,..., and *N*_*m*_, which is

$$ \sum_{x_{1}=1}^{N_{1}} \sum_{x_{2}=1}^{N_{2}} \dots \sum_{x_{m}=1}^{N_{m}} x_{j} P_{j} \prod \limits_{j \in J} \Pr(X_{j} = x_{j}), $$

is obviously no less than that with the original booking limits as long as *N*_*j*_≥*B*_*j*_ for all *j*∈*J*. However, each resource has its *ideal consumer level*, which is the maximum number of consumers that may be served with that resource in regular time. Once the number of arrived consumers requiring a resource exceed the ideal consumer level, for which we say *overage* occurs, overtime work is needed and consumer waiting cannot be avoided.

The *overage cost* under a booking limit combination *N*_1_,*N*_2_,..., and *N*_*m*_ may be expressed as follows. Let *K*_*i*_ be the ideal consumer level for resource *i* and *V* be an *n*×*m* matrix indicating the set-resource relationship, where *V*_*ij*_=1 if set *j* requires resource *i* and 0 otherwise. If *x*_*j*_ consumers who booked set *j* show up in a day, The *overage level* of resource *i* is

$$ \max\left\{\sum_{j \in J}V_{{ij}}x_{j} - K_{i}, 0\right\}. $$

When the overage level of a resource is positive, overtime work and consumer waiting incur some kind of cost for the physical examination center. Let *f*_*i*_(*z*) be the overage cost of resource *i* incurred by the center when the overage level is *z*. To reflect the practice of a physical examination center, *f*_*i*_(*z*) should be strictly increasing and strictly convex (so that the marginal overage cost is increasing). We should also have *f*(*z*)=0 for all *z*≤0. The total overage cost across all resources is thus

$$ \sum_{i \in I} f_{i}\left(\sum_{j \in J}V_{{ij}}x_{j} - K_{i}\right). $$

The center manager’s problem is to find a booking limit combination to best balance the expected revenue and expected overage cost. In this study, we follow the idea of [19, 20] to define the expected *net reward* as the expected revenue minus expected overage cost (the same concept is also used by [10] but called net profit therein). More precisely, the center manager chooses *N*_1_,*N*_2_,..., and *N*_*m*_ to maximize the expected net reward

$$ \sum_{x_{1}=1}^{N_{1}} \sum_{x_{2}=1}^{N_{2}} \dots \sum_{x_{m}=1}^{N_{m}} \prod \limits_{j \in J} \Pr(X_{j} = x_{j}) \pi(x_{1}, x_{2},..., x_{m}) $$

where

$$ \pi(x_{1}, x_{2},..., x_{m}) = \sum_{j \in J} x_{j} P_{j} - \sum_{i \in I} f_{i}\left(\sum_{j \in J}V_{{ij}}x_{j} - K_{i}\right), $$

is the net reward when *x*_*j*_ consumers show up to do set *j* with no late cancellation.

### Logistic regression, clustering, and Monte Carlo simulation

To apply the aforementioned overbooking optimization model, the only issue remains is to estimate Pr(*X*_*j*_=*x*_*j*_), the probability for *x*_*j*_ consumers to show up for set *j*. One naïve way is to collect historical booking and cancellation data for a period of time. If there are *q*_1_ late cancellations among *q*_2_ bookings, one may use $r = \frac {q_{1}}{q_{2}}$ to be the late cancellation probability for each booking. The random variable *X*_*j*_ then follows the binomial distribution with *N*_*j*_ trials and sucess probability 1−*r*. However, as different consumers may have different show-up rates, finer estimation is possible.

To better estimate Pr(*X*_*j*_=*x*_*j*_), it is believed that consumers booking set *j* may have different late cancellation probability. For the *k*th booking for set *j* in a day, let *r*_*jk*_ be her/his probability of late cancellation. As *r*_*jk*_ may depend on many factors, once historical data are collected, one may first construct a logistic regression model to find out variables having significant correlation with late cancellation. For the regression model, the dependent variable is 1 if a booking is late canceled or 0 otherwise. One may either input independent variables according to her/his belief and domain knowledge, manually removing insignificant independent variables, or using variable selection methods like forward stepwise selection, backward stepwise selection, etc. To facilitate clustering, it is suggested to factorize all continuous independent variables.

As long as factors significantly correlated with late cancellations are identified, one may use those factors to cluster historical bookings. For example, if all consumers are either male or female, and gender is identified to be a significant factor, we may create two clusters containing bookings made by male and female consumers, respectively. If whether the examination is conducted in winter (which is defined as, say, November, December, and January) is also significant, each of the two clusters may be further split into two smaller clusters according to the examination season. To estimate the late cancellation probability of the *k*th booking for set *j*, one may then first find the cluster it belongs to according to its attributes and use the proportion of bookings with late cancellation in that cluster to be the late cancellation probability *r*_*jk*_.

With *r*_*jk*_s estimated for all *N*_*j*_ bookings requiring set *j*, Pr(*X*_*j*_=*x*_*j*_) may now be calculated. Theoretically, if *N*_*j*_ is small, this probability may be calculated by completely enumerating all the $N_{j} \choose x_{j}$ combinations for *x*_*j*_ out of *N*_*j*_ consumers to show up and summing up their probabilities. When *N*_*j*_ is large enough, however, this method is impractical, and we resort to the following Monte Carlo simulation with *T* iterations. In the *t*th iteration, we generate the number of consumers who do not cancel late by the following steps. Firstly, we decide whether a particular consumer show up by comparing a uniform random number *R*∈[0,1] and her/his late cancellation rate *r*_*jk*_. The consumer is considering showing up without late cancellation if and only if *R*≥*r*_*jk*_. After doing this lottery for all the *N*_*j*_ consumers, we count the number of consumers who show up and denote it by $\hat {x}_{j}^{t}$. After *T* iterations are done, with {$\hat {x}_{j}^{1}, \hat {x}_{j}^{2}$,..., $\hat {x}_{j}^{t}$}, we estimate Pr(*X*_*j*_=*x*_*j*_) as

$$ \frac{\sum_{t=1}^{T} \mathbbm{1}\{{\hat{x}_{j}^{t} = x_{j}} \}}{T} $$

for all *x*_*j*_=0,1,...,*N*_*j*_, where

$$ \mathbbm{1}\{{\hat{x}_{j}^{t} = x_{j}} \} = \left \{ \begin{array}{ll} 1 & \text{if }\hat{x}_{j}^{t} = x_{j} \\ 0 & \text{otherwise} \end{array} \right.. $$

We keep increasing *T* until Pr(*X*_*j*_=*x*_*j*_) converges.

### Search for the optimal booking limits

Combining the mathematical model and estimated probabilities obtained in the previous two subsections, one may now optimize the booking limits for all sets. It is natural to evaluate the performance of three booking policies: *no overbooking* (the NO policy) *overbooking with uniform probability* (the OU policy), and *overbooking with clustering* (the OC policy). The NO policy allows no overbooking. Under policy OU, overbooking is allowed while all bookings are assumed to have same late cancellation probability estimated as the proportion of late cancellations in all historical bookings. A search for optimal booking limits is then needed. As for the OC policy, the late cancellation probability of a booking is estimated as the proportion of late cancellations in the cluster it belongs to. The optimized booking limits may thus be different under the two overbooking policies.

To search for optimal booking limits, first a search space must be defined by determining the *maximum allowable overage* for each resource, which should be set to a level so that overage above this level is not acceptable. For resource *i*, let *U*_*i*_ be the maximum allowable overage of resource *i*, a constraint

1 $$ \sum_{j \in J} V_{{ij}} N_{j} \leq K_{i} + U_{i}  $$

is added into the stochastic mathematical model to ensure that the overage level can never exceed *U*_*i*_. The search space is then defined as

$$ S = \left\{(N_{1},..., N_{m}) \left| \sum_{j \in J} V_{{ij}} N_{j} \leq K_{i} + U_{i} \forall i \in I\right.\right\}. $$

For the NO policy, *U*_*i*_ is set to 0 for all *i*∈*I* to ensure no overage with 100% probability.

Depending on the number of sets and the magnitudes of booking limits, one may either do a complete search or a greedy search. A complete search considers all booking limit combinations (*N*_1_,...,*N*_*m*_) in the search space *S*, i.e., satisfying all these constraints. The expected net rewards of all feasible combinations are evaluated, and the combination with the highest expected net reward is considered optimal and reported. If *m* is too large or *U*_*i*_s are too large, a complete search may not be finished in a reasonable amount of time. One may then resort to a greedy search. Starting from the combination (*N*_1_,*N*_2_,...,*N*_*m*_)=(0,0,...,0), i.e., the original booking limits, a greedy search runs several iterations. In each iteration, it evaluates the potential of each set by calculating the expected net reward resulted from increasing the booking limit of the set by one unit and keeping all other booking limits unchanged. The set with the highest potential is selected, the corresponding booking level is increased by 1, and then the next iteration starts. The greedy search stops when no increment brings in higher expected net reward or all increments lead to a combination outside the search space *S*. Note that the greedy search may not always obtain a truly optimal booking limit combination. When one applies it, one needs to study the optimality gap generated by the greedy search and modify the greedy search when necessary.

### Discrete-event simulation and performance evaluation

2 Once the optimal booking limit combination for a policy is obtained, we may evaluate it performance by calculating its expected net reward. As the real environment is actually uncertain, the calculation may be done by a discrete-event simulation. For the policy, we test its optimal booking limit combination by simulating bookings and late cancellations following the probability distributions estimated based on historical data. The simulation may be conducted in any time unit as one wants, say, day, week, month, or year. For example, suppose that one wants to evaluate the expected daily net rewards for a policy, she/he may generate bookings for a day following historical distributions. The first $N_{j}^{*}$ bookings will be accepted, where $N_{j}^{*}$ is the booking limit for set *j* in the optimal booking limit combination. Some of these accepted bookings will then be canceled late, where the occurrence of late cancellations also follows historical distributions. The realized revenue of each set and realized overage cost of each resource may then be combined to calculate the net reward for this day. The process may then be repeated multiple times, say, 50 times, for one to collect several daily net rewards. The average of these daily net rewards is then an estimate of the expected net reward brought by the optimal booking limit combination for this policy.

Using the discrete-event simulation described above, we may obtain the average net rewards and net reward distributions for the three booking policies. Their relative performance with respect to expected net rewards may then be compared, and statistical tests such as Tucky HSD test may then be conducted to test the significance of differences. Note that while the optimal booking limit combinations of the NO and OU policies are obtained by assuming a uniform late cancellation probability, in the discrete-event simulation the late cancellation probabilities should be the real ones, which are estimated by the ones used in the OC policy.

Finally, beside expected net rewards, other performance indicators may be also important. To name a few, the expected number of consumers served is a better indicator of healthcare service accessibility, and the expected total overage is more related to service quality. As these quantities are required during the calculation of the expected net reward, these indicators may also be calculated through the discrete-event simulation process.

## Results

To demonstrate how to develop an overbooking policy using the proposed way and evaluate its performance, we collaborate with a physical examination center in Taipei, Taiwan to conduct a case study.

### Overview of the physical examination center

Before our collaboration, the center does not adopt overbooking. The sum of booking limits for all sets, which is determined by center staffs using experience, is around 30. These spots are basically fully reserved everyday, and most of them are booked 180 days prior to the examination days, i.e., booked at the first day when reservation is open.

Appointment cancellations and rescheduling occur a lot everyday in the physical examination center. While most cancellations and rescheduling can be resolved by waiting for new appointments or moving later appointments to an earlier day to fill the vacancy, cancellations within seven days of the examination day are generally too late to have someone filling the spot. These cancellations are thus defined as late cancellations in this case study. In 2019, around 4% of bookings were canceled late, and late cancellations caused roughly 12,000,000 New Taiwan Dollar (NTD) losses in revenue and around 4% of examination opportunities wasted. Given that there are always many consumers waiting for examination, overbooking is considered.

The center serves three types of consumers: the general public, employees of companies having special contracts with the center, and government officials. In 2019, out of the total 12,390 bookings, 8,265 (66.7%), 2,966 (23.9%), and 1,159 (9.4%) consumers were of the three types, respectively. As the general public counts for the most bookings, and spots must be reserved to these companies and the government, in this case study we only focus on the general public, among which around 3.3% bookings were cancelled late.

### Data preprocessing and exploratory analysis

In 2019, in total 22 examination sets requiring eight resources were offered for consumers to self-select. The examination sets and their corresponding resources are in Table 13 in the appendix. Prices for the sets range roughly from 20,000 NTD to 100,000 NTD. Among the 22 sets, the top nine sets counted for more than 95% of the total bookings. To reduce computation complexity without sacrificing accuracy too much, we choose to consider only these nine sets. The resources that are under consideration is then limited to basic check, breast check, gastroenteroscope, magnetic resonance imaging (MRI), and diffusion weighted imaging (DWI). Furthermore, we aggregate the nine sets into four “aggregate sets” so that sets in the same aggregate set use the same resources. The sets, aggregate sets, and required resources are listed in Table 2. In the sequel, we call aggregate sets as set for simplicity. The picked sets and their corresponding booking percentages are put in the appendix.

**Table 2:** Aggregate sets, sets, and required resources

Aggregate	Set(s)	Resource				
Set		Basic	Breast	Gastroenteroscope	MRI	DWI
1	Male Basic Check Plus Colonoscopy	$\checkmark $		$\checkmark $		
2	Female Basic Check Above 40 Plus Colonoscopy	$\checkmark $	$\checkmark $	$\checkmark $		
	Female Basic Check Below 40 Plus Colonoscopy					
3	Male Exquisite Anticarcinogenic (with MRI)	$\checkmark $			$\checkmark $	
	Female Exquisite Anticarcinogenic (with MRI)					
4	Male Elite (with MRI + DWI)	$\checkmark $			$\checkmark $	$\checkmark $
	Female Elite (with MRI + DWI)					
	Male Advanced Anticarcinogenic (with MRI + DWI)					
	Female Advanced Anticarcinogenic (with MRI + DWI)					

Among the 8265 bookings, some consumers rescheduled their original booking to a new examination date. In this case, a booking is split into two bookings, where the first one’s cancellation date and the second one’s booking date are both set to be the date of rescheduling. 326 new bookings are thus created. After a data cleansing process whose details are presented in the appendix, 160 bookings with unrecoverable missing values are removed. The number of remaining bookings is now 8,431, among which 8,046 bookings are for the top nine sets aforementioned. These 8,046 bookings form the final data set for the regression analysis. The proportion of late cancellation is 3.34% in the final data set, slightly higher than 3.3% in the original 8265 bookings.

For each bookings, nine variables are recorded, including the consumer’s gender and age, dates of booking, examination, and cancellation (if applicable), the chosen examination set, etc. These original independent variables are listed in the first part of Table 3. We then create five derived independent variables, listed in the second part of the table, using the original ones. The last part of the table contains the dependent variable, late cancellation, which is derived by comparing the examination and cancellation dates.

**Table 3:** Variables

Variable	Description	Data type
Age	Age of the consumer	Numerical
Gender	Gender of the consumer	Categorical
Booking Date	The date when the consumer books an examination	Date
Booking Type	The way the consumer makes a reservation (in-person, online, or other)	Categorical
Examination Date	The date the consumer receives health examination	Date
Examination Set	The examination set chosen by the consumer	Categorical
Price	Price of the examination	Numerical
Group Size	Number of people booking together	Numerical
Cancellation Date	The date of cancellation (if applicable)	Date
Appointment Lead Time	Number of days between booking date and examination date	Numerical
Gastroscope	Whether the examination includes gastroscope or not	Categorical
Holiday Examination	1 if the examination is in a national holiday or 0 otherwise	Binary
Group Booking	1 if the group size is at least two or 0 otherwise	Binary
Last-minute Booking	1 if the appointment lead time is no greater than seven days or 0 otherwise	Binary
Late Cancellation	1 if canceled within seven days of the examination date or 0 otherwise	Binary

Before conducting the regression analysis, we try to find out important factors that are correlated with late cancellations through exploratory data analysis. To facilitate clustering of bookings, which will be conducted in later steps, various ways to divide numerical variables (such as age and price) and group date variables (such as examination date and booking date) are investigated. For example, from the raw data it is observed that January, February, August, November, and December have late cancellation probabilities higher than the overall average. We thus manually create several ways of grouping months, such as “November to February, July to August, others”, “November to January, August, others”, etc., and do regression with each way of grouping to see which one provides the best explanatory power. As the grouping “November to January, July to August, others” best explains late cancellations in our study, it is adopted in the final model. The grouping of other variables are also in a similar manner.

**Fig. 1:**
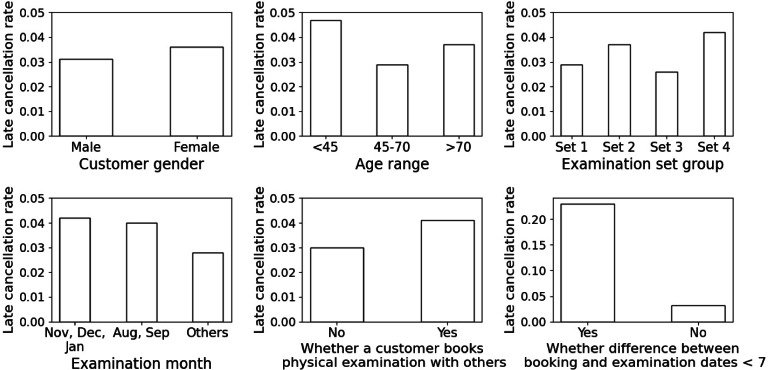
Visualization of late cancellation rates

After some exploration, four variables are worth reporting, including age interval, examination season, group booking, and last-minute booking. The visualization is provided in Fig. 1 with detailed numbers provided in Table 12 in the appendix. The plots and numbers regarding gender and examination sets are also provided just for reference.

The above process generates some managerial insights. First of all, consumers within the age interval 45 to 70 end up having lower late cancellation probability in average. This is not surprising, as middle-age people typically care about their health condition the most. Second, the average late cancellation probability is much higher in winter (November, December, and January) and summer (August, September) compared with the rest of the year. According to the staffs in the collaborating center, the huge number of events before New Year (both January 1 and the Chinese New Year) raises the late cancellation probability in winter. Similarly, events close to the end of summer vacation raise the probability in summer. Third, those booking with others have higher late cancellation probability in average than those booking individually. This can be due to the fact that when two or more consumers plan to go for physical examinations at the same day, they may all cancel together whenever one of them cannot make it. Finally, last-minute bookings, though seldom occur, have much higher late cancellation probability than usual bookings. The staffs also have no idea about the reason. Regardless of fitting one’s intuition or not, the exploratory analysis suggests us about variable creation and selection for regression model building.

### Late cancellation probability estimation

To identify significant factors that are correlated with late cancellation, we conduct logistic regression with late cancellation as the dependent variable. Three sets of independent variables are considered for our collaborating center. First, we consider those variables reported to be useful in literature (cf. Table 1) that we may collect, including age, gender, appointment lead time, examination season, and examination set. Continuous variables are factorized by categorizing bookings into interval following our exploratory data analysis. For the variable age, we create two intervals, namely between 45 and 70 or not. For appointment lead time, we create two levels, namely no greater than seven days (last-minute appointment) or not. For examination season, three levels are created, namely the winter season including November, December, and January, the summer season including August and September, and the off-peak season including the remaining seven months. Second, we input the three derived independent variables that seem to be effective in the exploratory analysis, including group booking, last-minute booking, and holiday examination (cf. Table 3. Whether these two sets of variables are statistically significant (with 0.05 as the threshold of *p*-value) is examined, and those insignificant variables are removed from the regression model. After this step, the remaining independent variables we have that are untested (such as price) are input as the third set of variables. A variable is kept in the model if it is statistically significant.

The above model building process results in the final regression model presented in Table 4. At the end, five factors are significant, including age interval, examination season, holiday examination, group booking, and last-minute booking. While intuitions obtained from exploratory data analysis (cf. Fig. 1 and Table 12) are mostly confirmed, gender and examination sets turn out to have no significant correlation with late cancellations. According to the regression report, the risk of late cancellations is higher if a consumer is too young (below 45) or too old (above 70), the examination is in winter or summer (especially winter), the examination is in a national holiday, the booking is a group booking, and the booking is done in the last minute.

**Table 4:** Regression report

Variables	Coefficient	Standard error	*z*	*p*-value
Constant	− 3.3203	0.112	− 29.518	≈0
Age Interval (level: [45,70])	− 0.4303	0.116	− 3.702	≈0
Examination Season (level: summer)	0.3711	0.135	2.754	0.006
Examination Season (level: winter)	0.4147	0.158	2.627	0.009
Holiday Examination (level: yes)	0.5702	0.311	1.836	0.066
Group Booking (level: yes)	0.2861	0.121	2.356	0.018
Lase-minute Booking (level: yes)	2.0522	0.325	6.321	≈0

According to the categorical values of the five significant factors, theoretically each booking may be clustered into one of 2×3×2×2×2=48 clusters. Nevertheless, because there are two few bookings that are in holidays or made in the last minute (cf. Table 12), these two factors are not used in clustering to avoid too sensitive estimation. Consequently, we obtain 2×3×2=12 clusters in total. The clusters and the proportion of bookings having late cancellations in each cluster are shown in Table 5. Unsurprisingly, the cluster of winter examination, group booking, and age not within 45 and 70 has the highest proportion of late cancellation (6.07%). On the contrary, the cluster of off-peak examination, individual booking, and age within 45 and 70 has the lowest proportion (1.81%). The highest proportion is about 3.5 times higher than the lowest one.

**Table 5:** Clusters of booking

Cluster	Examination season	Group booking	Age intervel: [45,70]	Number of bookings	Number of late cancellations	Late cancellation probability
1	Winter	Yes	Yes	583	20	3.43%
2	Winter	Yes	No	247	15	6.07%
3	Winter	No	Yes	842	31	3.68%
4	Winter	No	No	433	22	5.08%
5	Summer	Yes	Yes	236	10	4.24%
6	Summer	Yes	No	111	4	3.60%
7	Summer	No	Yes	578	19	3.29%
8	Summer	No	No	311	16	5.14%
9	Off-peak	Yes	Yes	835	32	3.83%
10	Off-peak	Yes	No	420	19	4.52%
11	Off-peak	No	Yes	2319	42	1.81%
12	Off-peak	No	No	1131	39	3.45%

The clustering result provides us finer estimation of the late cancellation probability of a booking. For each booking, we find the cluster it belongs to and use the historical late cancellation rate of the cluster as our estimated late cancellation probability. As an example, if a 38-year-old consumer makes an individual booking with no others in August, this booking will be clustered into cluster 8, and the estimated late cancellation probability will be 5.14%, rather than 3.34% as the overall historical late cancellation proportion.

It should be noted that, while in our study months should be categorized in to three groups (November to January, July to August, and others) to create the best model, it is by no means also the best for other studies. Different studies may have different research objectives, and customers in different regions may have different behaviors. Instead of simply using the ways of grouping adopted in our study, researchers are suggested to find their own ways to group months as well as other variables.

### Booking limit optimization

As we introduce in Section 4, we consider three booking policies, no overbooking (NO), overbooking with uniform probability (OU), and overbooking with clustering (OC). Recall that the five resources considered in this case study are basic check, breast check, gastroenteroscope, MRI, and DWI. For the NO policy, which represents the current policy prior to this study, the ideal consumer level for the five resources are *K*_1_=36,*K*_2_=36,*K*_3_=24,*K*_4_=4, and *K*_5_=2.

Both the NO and OU policies assume that all bookings have the same late cancellation probability, which is 3.34% in this study. Under the OC policy, however, the late cancellation probabilities are assumed to be different from cluster to cluster according to Table 5. As overbooking is not allowed under the NO policy, the maximum allowable overage *U*_*i*_=0 for all resource *i*∈*I*, and the search for an optimal booking limit combination is subject to the constraint in (1) with *K*_1_ to *K*_5_ as the right-hand-side values. By adopting the set-resource relationship recorded in Table 2, the five constraints may be listed as

$$\begin{array}{*{20}l} & N_{1} + N_{2} + N_{3} + N_{4} \leq 36, N_{2} \leq 36, \\ & N_{1} + N_{2} \leq 24, N_{3} + N_{4} \leq 4 \text{, and}\ N_{4} \leq 2. \end{array} $$

After discussions with center staffs, for the OU and OC policies, the maximum allowable overage of a resource is set to be 50% of the ideal consumer level of that resource, i.e., $U_{i} = \frac {1}{2}K_{i}$ for all *i*∈*I*. The right-hand-side values of these constraints therefore become 54, 54, 36, 6, and 3, respectively.

One may wonder why the maximum allowable overage rate 50% is much higher than 3.34%. To answer this, it should be noted that before the optimization is done, one has no way to know to what degree the original booking limits should be increased. While the proposed algorithm will search for optimal booking limits, one must first set a large enough search space for the algorithm not to miss the optimal booking limits. This is why a high maximum allowable overage rate is generally suggested. After all, setting $U_{i} = \frac {1}{2} K_{i}$ does not mean the optimal booking limits must result in a consumer level being equal to $\frac {3}{2}K_{i}$ for some resource *i*. The optimal booking limits will be determined by taking the overage costs into consideration. The higher the overage costs, the lower the optimal booking limits.

**Fig. 2:**
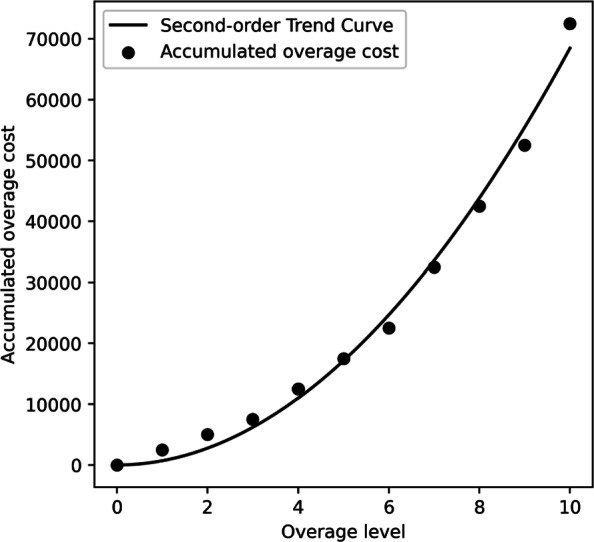
Overage Costs and the Second-order Trend Curve

It remains to determine the overage cost function *f*_*i*_(*z*) for each resource *i*∈*I* and overage level *z*. After discussions with center staffs, the marginal overage costs for the first ten units of overage are estimated by the collaborating center. In particular, the overage cost function, which is mainly based on overtime works from the perspective of the center, is set to be identical for all resources. It is estimated that each of the first three unit of overage incurs an overage cost of 2,500 NTD, each of the fourth to the six incurs a cost of 5,000 NTD, each of the seventh to the ninth incurs a cost of 10,000 NTD, and the tenth incurs a cost of 20,000 NTD. The accumulative overage cost of a resource is the sum of all marginal overage costs. For example, if for a resource the overage level is five, the total overage cost of that resource will be 2, 500×3+5, 000×2=17, 500 NTD. For overage levels over ten, center staffs find it too difficult to imagine and estimate. A second-order trend curve with zero intercept, *y*=18.802*x*+682.73*x*^2^, is thus generated using the accumulative overage costs for the first ten units (cf. Fig. 2). The accumulative overage cost of eleven and twelve units of overage may then be estimated as 82,871 NTD and 98,539 NTD, respectively. It is clear that the spillover effect of overage exists as the marginal cost is increasing in the overage level.

We are now ready to search for optimal booking limit combinations for the three booking policies. For this case study, the size of the search space is manageable, and thus complete search is adopted. After running through all feasible combinations, the search may return the one with the highest expected net reward for each policy. Note that as the late cancellation probabilities in different seasons are considered to be different in the OC policy, it is natural for the optimal combinations under the OC policy to differ in seasons. Table 6 lists the optimal combinations, where $N^{*}_{j}$ is the booking limit of resource *j* in the optimal combination. It may be observed that the booking limits are higher under the overbooking policies. Moreover, under the OC policy, $N^{*}_{2}$ is the lowest in the off-peak season. This is because that late cancellations are believed to occurs less likely in the off-peak season.

**Table 6:** Optimal booking limits under different policies

Policy	Season	$N^{*}_{1}$	$N^{*}_{2}$	$N^{*}_{3}$	$N^{*}_{4}$
NO	–	1	23	2	2
OU	–	1	35	3	3
OC	Winter	1	35	3	3
OC	Summer	1	35	3	3
OC	Off-peak	1	34	3	3

Note that a set of optimal booking limits must satisfy the resource constraint (1). For example, under the NO policy, the booking limits for the first two sets are $N_{1}^{*} = 1$ and $N_{2}^{*} = 23$. The constraint for resource 3 (*N*_1_+*N*_2_≤24) makes it infeasible to accept more consumers for these two sets. Similarly, the constraint for resource 4 (*N*_3_+*N*_4_≤4) disallows the physical examination center to accept more consumers for the last two sets. This explains why the optimal booking limits only sum to 28 rather than 36 (the maximum among *K*_*i*_+*U*_*i*_ for all *i*∈*I*).

### Performance evaluation and comparison

**Fig. 3:**
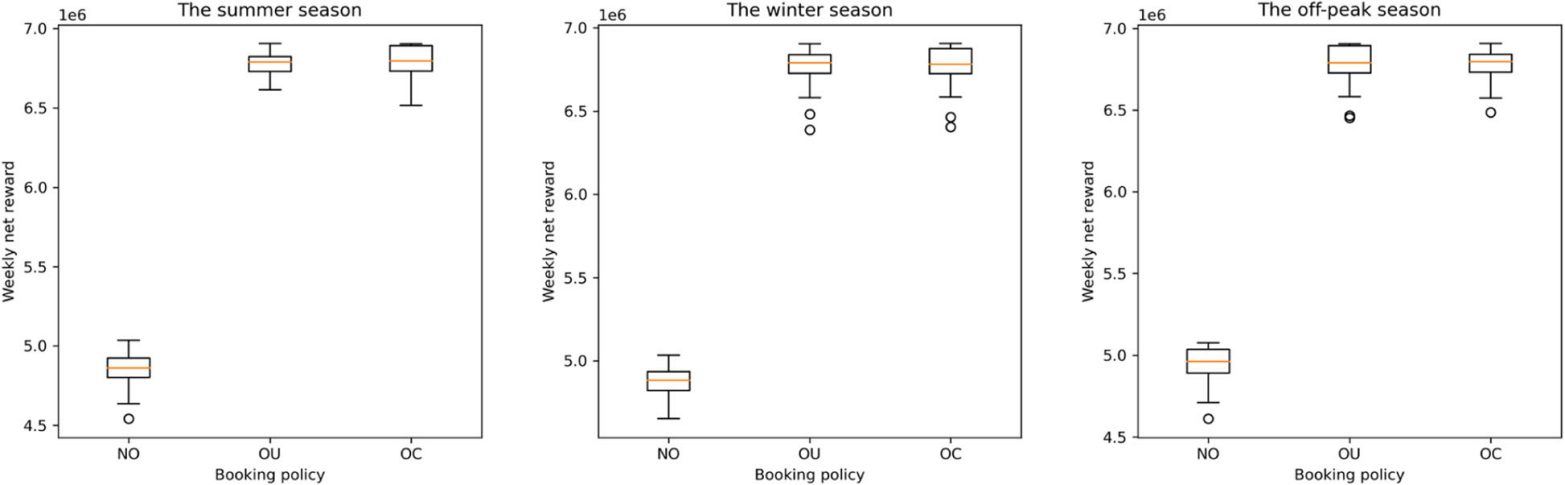
Boxplot of Simulated Fifty Weekly Net Rewards under Different Booking Policies

With the optimal booking limit combinations for the three policies, we apply the true late cancellation probabilities, which are estimated by the ones used in the OC policy, to evaluate the performance of each policy. The performance indicator we consider here is weekly expected net reward, which is estimated through discrete-event simulation. For each policy, we simulate bookings and late cancellations for 50 weeks. For each policy in each season, the average weekly rewards (in NTD) is presented in Table 7, and the distribution of the 50 average weekly rewards is visualized in the boxplot in Fig. 3.

**Table 7:** Average simulated weekly net reward

Policy	Weekly net reward		
	Summer	Winter	Off-peak
NO	4,903,168	4,892,003	4,943,680
OU	6,771,068	6,761,773	6,815,793
OC	6,781,929	6,784,008	6,809,758

From the simulation, we may see that overbooking indeed increases the average weekly net reward (as the NO-OU difference and NO-OC difference are both high), but whether clustering is beneficial for our collaborating center is unclear (as the difference between OU and OC is slim). Table 8, which contains the *p*-values reported by the Tukey HSD test about pairwise difference, confirms the observation. This somehow suggests that the probability estimation part does not generate significant benefit in this case study, which may be due to the fact that the late cancellation probabilities for all types of consumers are all low. Note that the *p*-value between OU and OC is the smallest in the winter season, in which the overall late cancellation probability is the highest among the three seasons. This suggests that probability estimation may still be beneficial in cases with higher overall late cancellation probability.

**Table 8:** Result of Tukey HSD test

Policy	*p*-value		
	Summer peak	Winter peak	Off-peak
NO vs. OU	0.001	0.001	0.001
NO vs. OC	0.001	0.001	0.001
OU vs. OC	0.562	0.175	0.824

A final remark should be made regarding the significant improvement on the expected net reward. According to the above experiments, adopting the optimal overbooking policy may help the physical examination center to increase its expected net reward by around 38%. It is important to note that, however, this is due to the fact that in the center currently the estimated overage costs are relatively low (compared to the prices of typical examinations). Once the overage costs are increased (e.g., due to an increase in overtime wages or a higher need to ensure consumer satisfaction), both the optimal booking limits and increased expected net reward should decrease.

**Fig. 4:**
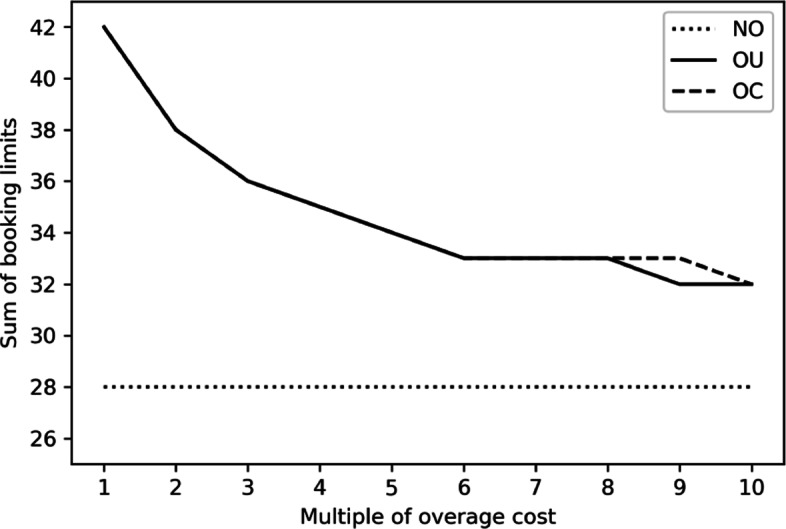
Impact of Overage Cost on the Optimal Booking Limits in Winter

**Fig. 5:**
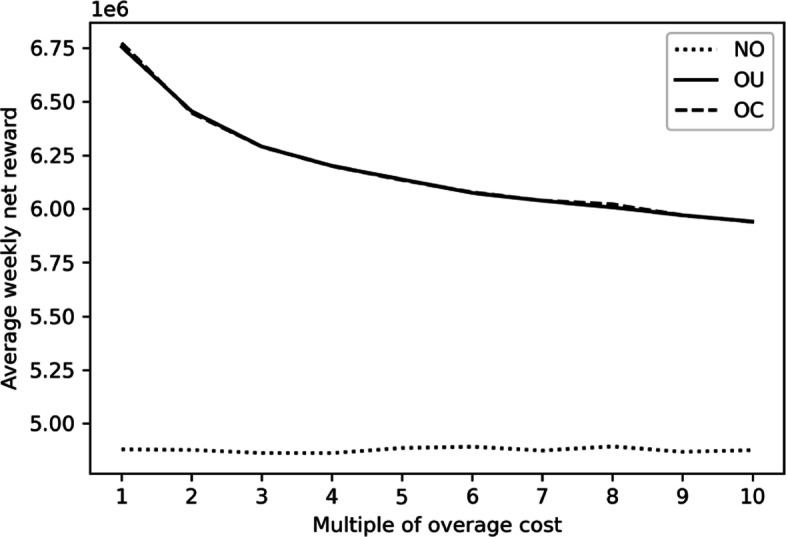
Impact of Overage Cost on the Expected Net Rewards in Winter

To see this, we repeat the discrete-event simulation in nine additional settings with the overage costs become two to ten times as much as the original ones. As the influence of costs is similar among the three seasons, we only demonstrate the analysis outcome in winter to save space. The impact of increasing the overage cost on the optimal booking limit combination and that on expected net reward are illustrated in Figs. 4 and 5. As the NO policy does not overbook, the NO policy is not affected. On the contrary, both the OU and OC policies overbook less as the overage costs become larger. The expected net reward also decreases in the overage costs. While the result is intuitive, it also demonstrate the applicability of our proposed solution in different scenarios.

## Discussion

Though the two overbooking policies are demonstrated to be effective in increasing expected net reward, it remains questionable how effective they may be in different scenarios. It is also important to ask whether they sacrifices too much in other perspectives. In this section, we conduct analysis with respect to overage cost estimation error, number of consumers served, and overage level to address these issues.

### Impact of the overage cost estimation error

**Fig. 6:**
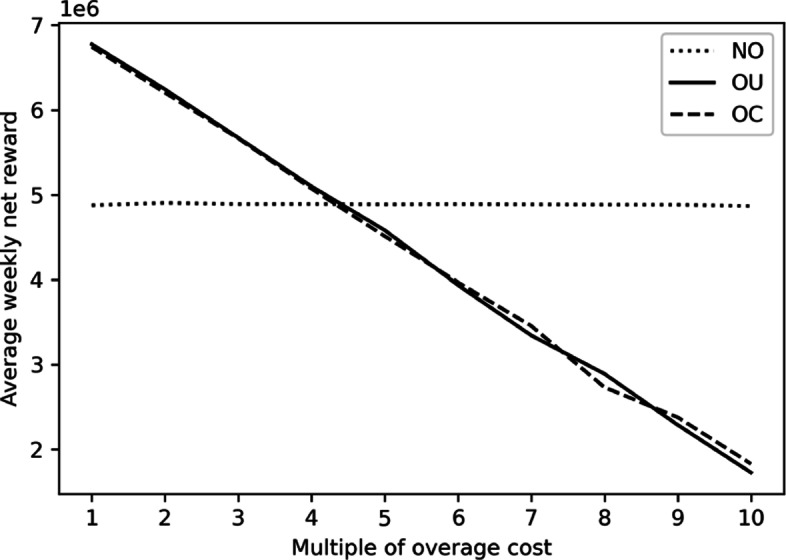
Expected Net Reward under Overbooking when Underestimating Overage Cost

In practice, it can be hard in many cases for a physical examination manager to estimate the cost by evaluating the loss brought by overtime works and patient waiting in monetary values. If the overage cost is greatly underestimated, overbooking may actually bring in a lower expected net reward. It is good if the benefit of overbooking is not too sensitive to the precision of overage cost estimation. Figure 6 depicts the result of a sensitivity analysis, in which the overage cost is actually multiple times of the one estimated by the decision maker adopting overbooking. For our collaborating center, even if the actual overage cost is four times of the estimated one, the expected net rewards under the two overbooking policies are still higher than that under no overbooking. Overbooking is thus quite applicable.

### More performance indicators

**Fig. 7:**
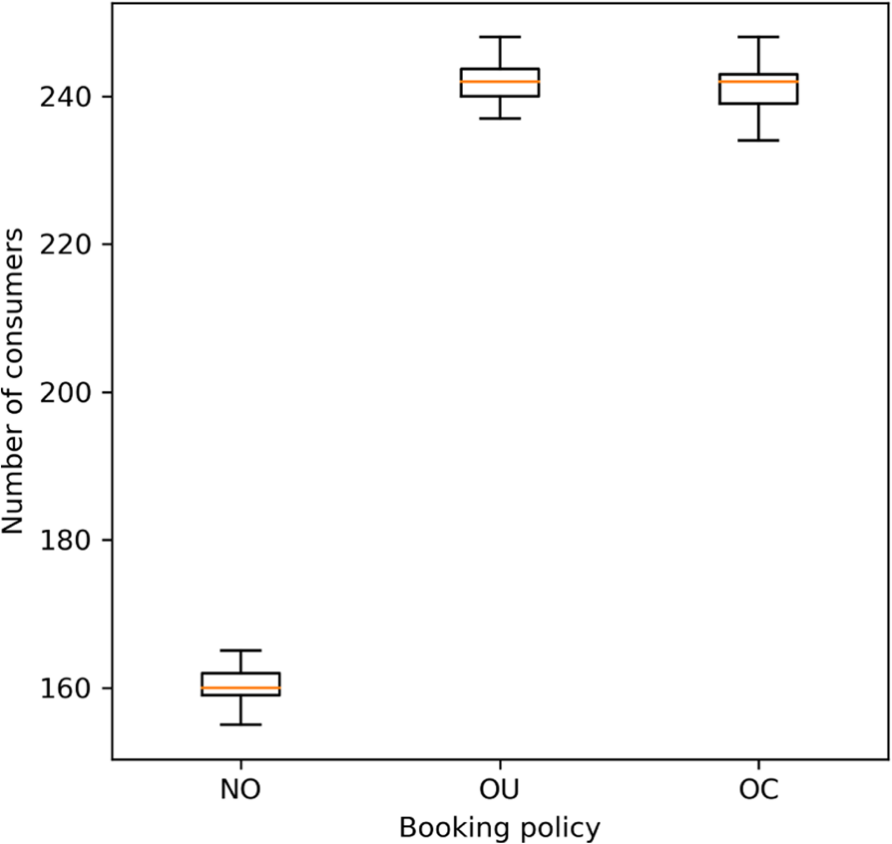
Number of Consumers Served per Week in Winter

We next investigate the number of customers served under the three policies. As shown in Fig. 7, though the number of consumers served is random under the two overbooking policies, such a number is consistently higher than that under no overbooking. In other words, the accessibility of healthcare resources is improved by overbooking as resource wastes are reduced in average.

**Fig. 8:**
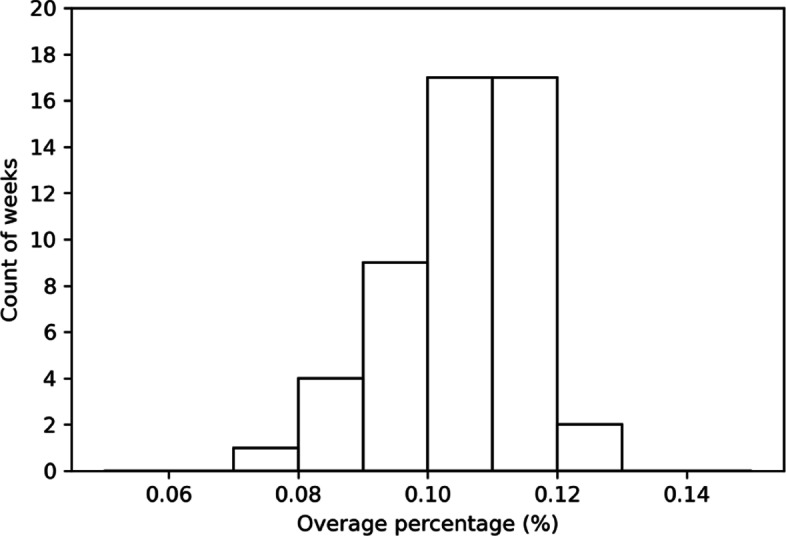
Overage percentages under the OC policy in Winter

While overbooking is helpful in enhancing expected net reward and healthcare resource accessibility, it unfortunately creates overage, which results in patient waiting and overtime works. Even if the formula of net reward already include overage cost, it is still important to put an eye on the degree of overage. For each resource in a period of time, let the *overage percentage* be the overage level divided by the number of consumers served. According to Fig. 8, which illustrates the distribution of the 50 weekly overage percentages under the OC policy in winter produced by the discrete-event simulation, in average roughly 10–11% of consumers served are considered overage. Considering the fact that the increase on expected reward is about 38% (cf. Table 7), the benefit seems to be large enough to compensate the detriment.

**Fig. 9:**
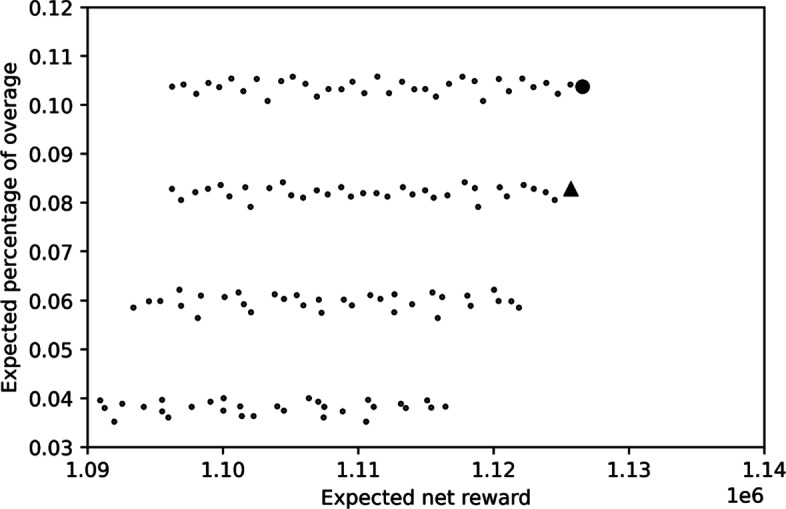
Overage percentages under the OC policy in Winter

In many case, increasing the expected net reward means to overbook more, which means creating more overage in expectation. Facing the trade-off between expected net reward and expected overage percentage, the concept of Pareto frontier may help. In Fig. 9, each circle is plotted according to the expected net reward and expected overage percentage resulted from a booking limit combination of the OC policy in winter. The optimal booking limit combination, which brings in the highest expected net reward, is plotted as the only solid circle. While this combination is the best along the dimension of expected net reward, it is not as good as many other combinations along the dimension of expected overage percentage. If the expected overage percentage is considered too high to be acceptable, all the combinations that have been examined during the search process may serve as alternatives. Suppose that there is a maximum allowable expected overage percentage, say, 7%, one may turn to choose the combination plotted as the only solid square, which generates the highest expected net reward than all combinations having low enough expected overage percentage.

In general, a combination is called Pareto-efficient if there is no other combination having higher expected net reward and lower expected overage percentage than it. Our proposed solution may report all Pareto-efficient combinations identified during the search process to a physical examination manager in practice. A booking limit combination may then be selected according to the manager’s decision criterion. The performance indicator expected overage percentage may also be substituted by expected overage level, expected overage cost, etc., to fit the need of a practitioner.

## Conclusion

In this study, overbooking for physical examination considering the relationship between examination sets and resources is studied. A stochastic mathematical programming model is formulated for expected net reward optimization, a way of late cancellation probability estimation is developed with logistic regression, Monte Carlo simulation, and clustering, and a way of performance evaluation is constructed through discrete-event simulation. With the help of a collaborating physical examination center in Taiwan, we conduct a case study to demonstrate the benefit of applying the proposed methods. In short, overbooking does help under the circumstance that late cancellation causes great loss. The higher the cancellation rate, the greater improvement on the expected reward.

This study is certainly limited and may be improved in several directions. First, probability estimation may be improved by using more relevant features. For example, an owner of a physical examination center in many cases also own clinics or hospitals. It may be promising to use a consumer’s records in the clinic/hospital to estimate its late cancellation probability. Second, while the search space of booking limit combinations is small in this study, it may be quite big in general and calls for the need of a heuristic search. Developing an effective and efficient heuristic search algorithm with rigorous performance evaluation can also be valuable. In particular, as an overbooking problem is to determine the capacity level facing uncertain demand, it shares the same characteristics with the well-known newsvendor model in the operations management literature [34, 35].^1^ While the set-resource relationship, integer constraints, and nonlinearity make the closed-form critical ratio formula cannot be directly applied, when a good heuristic algorithm is needed, algorithms solving variants of newsvendor problems are certainly great references. Finally, while in this study we do not consider interventions to reduce late cancellation probabilities, integrating overbooking with costly interventions may generate fruitful results.

## Appendix a: detailed data cleansing process

Because the impact of appointment cancellation and rescheduling request is similar, we view a rescheduling request as a cancellation combined with a new appointment. Therefore, for a booking with *N* bookings of changing examination dates, we break them down into *N*+1 new bookings, and for the second to last new bookings, the booking date is the cancellation date of the previous booking.

As an example, consider a booking made on February 3 asking for an examination on August 8. On April 7, the consumer asked for rescheduling to September 3. On June 2, the consumer further asked for rescheduling to October 15. The two rescheduling requests make the original booking be split into three bookings shown in Table 9.

We make some judging criteria of labeling rescheduling requests after discussing with physical examination center. First, Date exchanging between two consumers will not be viewed as changing date since the request will not cause losing on reward. Second, date changing caused by unpredictable factors, such as natural disasters or sudden closing of physical examination center, will not be viewed as rescheduling since that could not be controlled by the consumer. Third, since every set requires different resources, and changing sets will lead to additional administration costs, changing examination sets will be viewed as changing. However, the request of changing a single examination item will not be counted into changing.

There are some missing data in the raw data set. In very few cases, the date when consumers requested to change the examination dates are missing due to the human error of staffs. In these cases, we consider the average of the original booking date and the new examination date as the booking date of the new booking. After the process, we recover most missing data, but 160 bookings are still unrecoverable.

**Table 9:** Bookings after splitting

Booking date	Examination date	Cancellation date
2019-2-3	2019-8-5	2019-4-7
2019-4-7	2019-9-3	2019-6-2
2019-6-2	2019-10-15	N/A

After cleansing and splitting, there are 8,431 bookings, roughly 2% more than the original number of citizen bookings in the raw data. As mentioned, we only consider the top nine sets in this study. Thus, the number of bookings further decreases to 8,046. Furthermore, the late cancellation rate is 3.3% in the original data and 3.34% in the cleansed data.

## Appendix b: supplemental data

Table 10 lists the variables that are mentioned in other papers saying to have influence on the late cancellation rate but not used in this study.

**Table 10:** Factors considered in the literature

	[24]	[28]	[25]	[30]	[26]	[31]	[29]	[3]	[6]
Previous failure to attend	v						v	v	
Traffic convenience of the hospital	v						v		
Patient’s race		v			v		v		
Patient distress			v						
Patient opinion concering referal			v						
Patient’s health insurance type				v	v			v	v
Patient continuity of care				v					
Emotional barrier						v			
Concern about disrespect by the hospital						v			
Lack of understanding of the scheduling system						v			
Provision of cell phone number							v		
Type of patient (new/old)								v	
Booking confirmation/reminder service								v	
Type of booking								v	
Day of week								v	
Time of day								v	
Weather								v	

**Table 11:** Top nine examination sets

Set name	Bookings	Percentage
Male Basic Check Plus Colonoscopy	3068	36.4%
Female Basic Check Above 40 Plus Colonoscopy	2754	32.7%
Female Basic Check Below 40 Plus Colonoscopy	760	9.0%
Male Exquisite Anticarcinogenic (with MRI)	315	3.7%
Female Exquisite Anticarcinogenic (with MRI)	333	3.9%
Male Elite (with MRI and DWI)	387	4.6%
Female Elite (with MRI and DWI)	233	2.8%
Male Advanced Anticarcinogenic (with MRI and DWI)	108	1.3%
Female Advanced Anticarcinogenic (with MRI and DWI)	88	1.0%
Others	385	4.6%
Total	8431	100.0%

Table 11 provides a list of the top nine examination sets and their corresponding booking percentages among all sets.

Table 12 provides a list of the numbers of bookings, numbers of late cancellations, and late cancellation probabilities under different clustering categories.

**Table 12:** Late cancellation probability in categories

Variable	Category	Bookings	Late cancellations	Late cancellation probability
Gender	Male	3,875	119	3.1%
	Female	4,171	150	3.6%
Examination Set	Set 1	3,068	88	2.9%
	Set 2	3,514	130	3.7%
	Set 3	648	17	2.6%
	Set 4	816	34	4.2%
Age Interval	<45	1,666	78	4.7%
	45-70	5,393	154	2.9%
	>70	987	37	3.7%
Group Booking	No	5,614	169	3.0%
	Yes	2,432	100	4.1%
Last-minute Booking	Yes	47	11	23.0%
	No	7,999	258	3.2%
Season	Winter	2,105	88	4.2%
	Summer	1,236	49	4.0%
	Others	4,705	132	2.8%
Total		8,046	269	3.3%

**Table 13:** Examination sets and required resources

Examination Set	Description	Resources
Male Basic Check	The only difference between these two sets is the depth of the enteroscopy check. (sigmoid colon or the whole large intestine)	N/A
Male Basic Check Plus Colonoscopy		Gastroenteroscope
Female Basic Check Below 40	The differences between ages is the breast check. Breast ultrasound for female below 40 while mammography for the other.	Breast
Female Basic Check Above 40		Breast
Female Basic Check Below 40 Plus Colonoscopy		Breast + Gastroenteroscope
Female Basic Check Above 40 Plus Colonoscopy		Breast + Gastroenteroscope
Male Exquisite Anticarcinogenic (with MRI)	The differences between Exquisite Advanced Anticarcinogenic is the magnetic resonance imaging (MRI). They both include normal MRI. While the diffusion weighted imaging (DWI) is added into the second one, which can help distinguish malignant tumor from benign tumor.	MRI
Female Exquisite Anticarcinogenic (with MRI)		MRI
Male Advanced Anticarcinogenic (with MRI + DWI)		MRI + DWI
Female Advanced Anticarcinogenic (with MRI + DWI)		MRI + DWI
Male Elite (with MRI + DWI)	The Elite is the Advanced Anticarcinogenic plus the coronary angiogram.	MRI + DWI
Female Elite (with MRI + DWI)		MRI + DWI
Male Exquisite Cardiac	The Advanced Cardiovascular is the Exquisite Cardiac plus head MRI and cardia echo.	Cardia Echo
Female Exquisite Cardiac		Cardia Echo
Male Advanced Cardiovascular		Cardia Echo + Cerebrum
Female Advanced Cardiovascular		Cardia Echo + Cerebrum
Male Gastroenteroscope	The six examination set is mainly for the government employee. They can choose one from gastroenteroscope, computed tomography coronary angiography (CTCA) or breast computed tomography (CT).	Gastroenteroscope
Male Computed Tomography Coronary Angiography		Cardia Echo
Male Breast Computed Tomography		CT
Female Gastroenteroscope		Gastroenteroscope
Female Computed Tomography Coronary Angiography		Cardia Echo
Female Breast Computed Tomography		CT

Table 13 provides a list of all the examination sets and their corresponding valuable resources.

## Data Availability

The data set used in this study consists of transactions from individual consumers. Given that these are private data shared only by individual consumers and our collaborating physical examination center, we intend not to share this data set publicly.

## Abbreviations

NTD: New Taiwan dollar
MRI: Magnetic resonance imaging
DWI: Diffusion weighted imaging
CT: Computed tomography
NO policy: The no-overbooking policy
OU policy: The overbooking policy
OC policy: The overbooking with probability estimation by clustering policy
Tukey HSD test: Tukey honestly significant difference test

## Footnotes

1 We thank an anonymous reviewer for pointing this out.
